# Supplementary material for: MetaRibo-Seq measures translation in microbiomes
Source: Nat Commun. 2020 Jun 29;11:3268. doi: 10.1038/s41467-020-17081-z (PMC7324362; doi:10.1038/s41467-020-17081-z)
Supplement: Supplementary file 10 — Supplementary Data 7 [file 41467_2020_17081_MOESM10_ESM.zip › File2/Confidence_High_Taxonomy/58072_out.krona.html]

Javascript must be enabled to view this page.

members
magnitude
magnitudeUnassigned
count
unassigned
taxon
rank

58072\_out

201

2
superkingdom
201

1239
201
phylum
2

SRS017916\_contig\_number\_contig-100\_13535.88833SRS048060\_contig\_number\_contig-100\_7145.47174

1
class
526524

order
1
526525

1
family
128827

1
genus
1573535

1735
species
1

SRS098644\_contig\_number\_35326

class
198
186801

186802
order
198
8

SRS014948\_contig\_number\_contig-100\_14015.14016SRS016335\_contig\_number\_23028SRS019968\_contig\_number\_contig-100\_3543.40868SRS021219\_contig\_number\_contig-100\_20745.53371SRS098717\_contig\_number\_6939SRS098881\_contig\_number\_7523SRS145308\_contig\_number\_contig-100\_19237.19238SRS150029\_contig\_number\_contig-100\_26080.70411

8
family
541000


SRS065176\_contig\_number\_8078
species
1
39492

1263
genus
6

2293151

SRS015782\_contig\_number\_5425SRS019178\_contig\_number\_contig-100\_348.35068SRS1055049\_contig\_number\_contig-100\_15140.55275SRS893231\_contig\_number\_contig-100\_283.28864
species
4


SRS1041146\_contig\_number\_contig-100\_1021.1022
species
1
46228

species
1

SRS142503\_contig\_number\_45476
2292251

292632
1
genus

1897022
1
species

SRS148874\_contig\_number\_13701

186803
176
family
11

SRS015217\_contig\_number\_contig-100\_810.144385SRS016954\_contig\_number\_contig-100\_37891.67029SRS019030\_contig\_number\_19141SRS019601\_contig\_number\_3375SRS053335\_contig\_number\_contig-100\_4954.4954SRS1041031\_contig\_number\_27164SRS105082\_contig\_number\_13109SRS1054716\_contig\_number\_16717SRS148091\_contig\_number\_13261SRS148159\_contig\_number\_55637SRS893380\_contig\_number\_contig-100\_3175.3176

6
genus
1506577

29361
species
6

SRS013940\_contig\_number\_199SRS021153\_contig\_number\_1787SRS042966\_contig\_number\_contig-100\_15345.56414SRS147022\_contig\_number\_14098SRS147271\_contig\_number\_34654SRS893383\_contig\_number\_contig-100\_2402.111880


SRS077454\_contig\_number\_19048
species
1
1952145

13
genus
2569097


SRS017103\_contig\_number\_42149SRS017701\_contig\_number\_5588SRS019910\_contig\_number\_contig-100\_4195.61401SRS024009\_contig\_number\_contig-100\_7686.46057SRS046369\_contig\_number\_contig-100\_4744.78655SRS048870\_contig\_number\_contig-100\_1404.45169SRS056259\_contig\_number\_10289SRS074670\_contig\_number\_contig-100\_4950.4950SRS077552\_contig\_number\_1256SRS103987\_contig\_number\_13564SRS104485\_contig\_number\_contig-100\_10584.10584SRS144753\_contig\_number\_contig-100\_16860.16861SRS146812\_contig\_number\_55728
species
13
39488

1
genus
2039240

160404
1
species

SRS104400\_contig\_number\_53876

1898203

SRS055982\_contig\_number\_363
1
species

189330
32
genus


SRS143523\_contig\_number\_15765SRS149181\_contig\_number\_23087SRS893170\_contig\_number\_10982
3
species
88431

39486

SRS011405\_contig\_number\_18408SRS013158\_contig\_number\_contig-100\_218.40235SRS014923\_contig\_number\_43927SRS015065\_contig\_number\_33683SRS016267\_contig\_number\_contig-100\_221.83587SRS020233\_contig\_number\_59049SRS042284\_contig\_number\_2962SRS043001\_contig\_number\_8554SRS045244\_contig\_number\_7183SRS045826\_contig\_number\_22733SRS049402\_contig\_number\_contig-100\_375.117097SRS049896\_contig\_number\_34442SRS049959\_contig\_number\_34334SRS050925\_contig\_number\_37690SRS053214\_contig\_number\_19531SRS062427\_contig\_number\_10409SRS062654\_contig\_number\_25216SRS075078\_contig\_number\_contig-100\_2307.183850SRS104311\_contig\_number\_44245SRS1055022\_contig\_number\_25348SRS1055034\_contig\_number\_5625SRS1055043\_contig\_number\_contig-100\_1310.151559SRS1055083\_contig\_number\_12449SRS143598\_contig\_number\_17324SRS144362\_contig\_number\_contig-100\_190.259840SRS145497\_contig\_number\_449SRS147346\_contig\_number\_29911SRS148511\_contig\_number\_13300SRS893369\_contig\_number\_10311
species
29

1
genus
1407607

1150298

SRS016495\_contig\_number\_contig-100\_3023.3023
1
species

572511
38
genus


SRS046502\_contig\_number\_13718SRS140492\_contig\_number\_34185
2
species
765821

40520

SRS013098\_contig\_number\_23911SRS014613\_contig\_number\_1825SRS015431\_contig\_number\_16628SRS016095\_contig\_number\_31478SRS019068\_contig\_number\_3077SRS023715\_contig\_number\_23560SRS024331\_contig\_number\_34160SRS046717\_contig\_number\_3309SRS051031\_contig\_number\_27139SRS054590\_contig\_number\_1639SRS062701\_contig\_number\_5868SRS063190\_contig\_number\_15269SRS064973\_contig\_number\_2693SRS065397\_contig\_number\_13640SRS077502\_contig\_number\_8226SRS097958\_contig\_number\_21672SRS1041033\_contig\_number\_29887SRS1041091\_contig\_number\_16947SRS1041142\_contig\_number\_9361SRS144537\_contig\_number\_5021SRS147139\_contig\_number\_44493SRS148253\_contig\_number\_contig-100\_159.99982SRS149879\_contig\_number\_25625SRS892882\_contig\_number\_10349SRS971276\_contig\_number\_28348
25
species

33038
species
8

SRS014287\_contig\_number\_18859SRS016989\_contig\_number\_10117SRS018817\_contig\_number\_19067SRS020394\_contig\_number\_1670SRS044535\_contig\_number\_17289SRS077294\_contig\_number\_8886SRS098655\_contig\_number\_12227SRS143342\_contig\_number\_21583

1
species

SRS017433\_contig\_number\_18285
33039

2292990

SRS014235\_contig\_number\_48461SRS143895\_contig\_number\_40966
2
species

genus
54
841

3
species

SRS050026\_contig\_number\_12493SRS142712\_contig\_number\_5275SRS147377\_contig\_number\_12835
360807

2293137
50
species

SRS011084\_contig\_number\_30442SRS013965\_contig\_number\_39330SRS014683\_contig\_number\_19509SRS014855\_contig\_number\_contig-100\_2108.2109SRS014979\_contig\_number\_33547SRS015578\_contig\_number\_36721SRS015663\_contig\_number\_contig-100\_2692.176589SRS015694\_contig\_number\_13381SRS015854\_contig\_number\_23217SRS016203\_contig\_number\_8931SRS018623\_contig\_number\_2370SRS018836\_contig\_number\_5142SRS018936\_contig\_number\_contig-100\_463.47561SRS019267\_contig\_number\_95SRS043768\_contig\_number\_32888SRS043841\_contig\_number\_3222SRS045713\_contig\_number\_contig-100\_1567.99737SRS050752\_contig\_number\_contig-100\_597.115640SRS050941\_contig\_number\_20703SRS053649\_contig\_number\_13434SRS054905\_contig\_number\_500SRS057478\_contig\_number\_1640SRS058770\_contig\_number\_1877SRS063040\_contig\_number\_46927SRS063370\_contig\_number\_2570SRS063518\_contig\_number\_31447SRS064276\_contig\_number\_42949SRS076804\_contig\_number\_5459SRS077392\_contig\_number\_39102SRS097920\_contig\_number\_31417SRS098827\_contig\_number\_9001SRS1041090\_contig\_number\_1377SRS104327\_contig\_number\_contig-100\_906.907SRS104636\_contig\_number\_31019SRS104975\_contig\_number\_19539SRS105153\_contig\_number\_30302SRS1054691\_contig\_number\_6472SRS142599\_contig\_number\_27893SRS142890\_contig\_number\_42906SRS143085\_contig\_number\_2443SRS143876\_contig\_number\_41259SRS144135\_contig\_number\_27911SRS144183\_contig\_number\_contig-100\_623.170556SRS146813\_contig\_number\_25114SRS148196\_contig\_number\_31096SRS148424\_contig\_number\_contig-100\_1655.187277SRS149784\_contig\_number\_28446SRS893252\_contig\_number\_5229SRS893256\_contig\_number\_2623SRS893373\_contig\_number\_contig-100\_1965.141064


SRS019445\_contig\_number\_16036
1
species
1262947

33042
17
genus

410072
species
15

SRS012273\_contig\_number\_27801SRS022071\_contig\_number\_17820SRS024663\_contig\_number\_10521SRS042628\_contig\_number\_8776SRS043701\_contig\_number\_3473SRS047433\_contig\_number\_contig-100\_14905.14906SRS065504\_contig\_number\_32648SRS1041038\_contig\_number\_9236SRS1041132\_contig\_number\_2037SRS1041136\_contig\_number\_20558SRS1055076\_contig\_number\_contig-100\_896.99734SRS142980\_contig\_number\_937SRS893270\_contig\_number\_22421SRS893295\_contig\_number\_8645SRS893378\_contig\_number\_7395

116085

SRS020869\_contig\_number\_20194SRS075821\_contig\_number\_3865
species
2

1769710
1
genus

1653434
species
1

SRS023971\_contig\_number\_4721

1
family
186806

1730
genus
1

species
1

SRS050998\_contig\_number\_13965
142586

family
5
31979

genus
5
1485


SRS046068\_contig\_number\_contig-100\_960.35706SRS053356\_contig\_number\_46594SRS077194\_contig\_number\_30631SRS147977\_contig\_number\_11706
species
4
59620

2293002

SRS048164\_contig\_number\_30331
species
1
